# Progression of LAMP as a Result of the COVID-19 Pandemic: Is PCR Finally Rivaled?

**DOI:** 10.3390/bios12070492

**Published:** 2022-07-06

**Authors:** Cassidy Mannier, Jeong-Yeol Yoon

**Affiliations:** Department of Biomedical Engineering, the University of Arizona, Tucson, AZ 85721, USA; cmannier17@email.arizona.edu

**Keywords:** SARS-CoV-2, RT-PCR, RT-LAMP, loop-mediated isothermal amplification, rapid antigen test, nucleic acid amplification test (NAAT)

## Abstract

Reflecting on the past three years and the coronavirus disease 19 (COVID-19) pandemic, varying global tactics offer insights into the most effective public-health responses. In the US, specifically, rapid and widespread testing was quickly prioritized to lower restrictions sooner. Essentially, only two types of COVID-19 diagnostic tests were publicly employed during the peak pandemic: the rapid antigen test and reverse transcription polymerase chain reaction (RT-PCR). However, neither test ideally suited the situation, as rapid antigen tests are far too inaccurate, and RT-PCR tests require skilled personnel and sophisticated equipment, leading to long wait times. Loop-mediated isothermal amplification (LAMP) is another exceptionally accurate nucleic acid amplification test (NAAT) that offers far quicker time to results. However, RT-LAMP COVID-19 tests have not been embraced as extensively as rapid antigen tests or RT-PCR. This review will investigate the performance of current RT-LAMP-based COVID-19 tests and summarize the reasons behind the hesitancy to embrace RT-LAMP instead of RT-PCR. We will also look at other LAMP platforms to explore possible improvements in the accuracy and portability of LAMP, which could be applied to COVID-19 diagnostics and future public-health outbreaks.

## 1. Introduction: Hesitancy to Embrace LAMP in SARS-CoV-2 Diagnostics

As the severe acute respiratory syndrome coronavirus 2 (SARS-CoV-2) pandemic comes to an endemic phase, our society enters a consideration period regarding our actions and responses, both successful and not. Though we have suffered a significant loss, we now have an opportunity to learn and better prepare for future public-health crises. In terms of preventative measures, two major techniques lead to outbreak control: strict lockdowns and widely available rapid testing [1]. In the US, specifically, prevention mandates ended far sooner than in other countries, and widespread COVID-19 testing became heavily relied on [2,3,4,5]. Specifically, the rapid antigen test and RT-PCR test were the two primary testing options [6,7,8,9,10,11]. In the case of RNA viruses, such as SARS-CoV-2, RNA must be converted to complementary DNA. This conversion is conducted by the additional reverse-transcription step before the amplification. However, with clear disadvantages of each test—the inaccuracy of the rapid antigen test, and the long assay time and cost of the RT-PCR test, perhaps other testing options should have been considered [12]. At the peak of the pandemic, time-to-results of PCR tests reached up to two weeks in some areas, due to issues with sample delivery and logistics [13,14,15]. LAMP offers the sensitivity and specificity of nucleic-acid-based tests, while maintaining a short assay time and simplicity of the assay [16]. LAMP requires only one temperature, while PCR typically requires two to three temperatures. While we can expect the same for RT-LAMP-based SARS-CoV-2 diagnostics, implementation has not been widespread during the pandemic’s peak. Such hesitancy may be attributed to the relatively lower accuracy than PCR and the relative complexity of the device over the rapid antigen tests. However, in recent years, and specifically during the pandemic, LAMP research has surged, leading to significant improvements in these areas. This surge will be further analyzed in Section 2.

PCR is considered the “gold standard” and has been, by far, the preferred approach for COVID-19 testing [9]. However, PCR needs more sophisticated laboratory equipment such as a thermocycler, expensive machinery that can rapidly change the temperature for each cyclical stage (denaturation, annealing, and extension) [17]. As such, PCR tests are rarely conducted at the point of care, since, rather, samples must be sent to laboratories, lengthening the time to results. In LAMP, on the other hand, four to six primers (forward inner and outer primers, as well as backward inner and outer primers) are implemented to form “dumbbell-like” loops of continuous isothermal deoxy-/ribonucleic acid (DNA/RNA) replication. The process removes denaturation and annealing temperatures, allowing for a single “extension” temperature (thus, “isothermal”) [18]. When paired with fluorescence/colorimetry/turbidity measurements, or gel electrophoresis, it can detect the even minute presence of pathogens. LAMP reactions can be conducted at the point of care with accessible, affordable heating equipment, such as hot plates, combined with simple visual-detection methods. In the case of quantitative real-time analysis (in which a factor such as fluorescence is constantly monitored as the reaction takes place), the time to results (starting at sample collection) can be under an hour and a very small number of targets [19,20] can be detected, even in more complex samples such as blood [21].

Despite eliminating secondary-testing locations, expensive equipment, and longer wait times, LAMP remains in the shadow of PCR. There is a need for conservative sites for four–six primers, which can be challenging for highly diverse viruses. In addition, LAMP shows inferior accuracy: compared to PCR, LAMP is more prone to false positives, resulting from primer-dimers and non-specific amplification [21,22,23].

Numerous research groups have attempted to tackle this issue, yielding a wide range of techniques. For example, many research groups have utilized oligonucleotide-strand-displacement (OSD) probes, fluorophore-labeled nucleic-acid strands that are sequence-specific, rather than relying on non-specific-reaction byproducts [24,25,26,27,28]. Both microfluidics [29,30,31] and emulsion [32] reactions have also been applied to LAMP diagnostics to compartmentalize the reactions, once again improving accuracy. Adding dimethyl sulfoxide (DMSO) to the reaction has also been proven to increase LAMP’s analytical specificity and sensitivity [33] and clinical specificity and sensitivity [34,35]. In addition, in the recent years of the SARS-CoV-2 pandemic, LAMP-centered research has especially exploded. This begs the question: will LAMP ever reach the potential of PCR diagnostics, and, if so, will the medical community ever embrace it?

## 2. Post-Pandemic Surge in LAMP-Diagnostics Research

**Figure 1:**
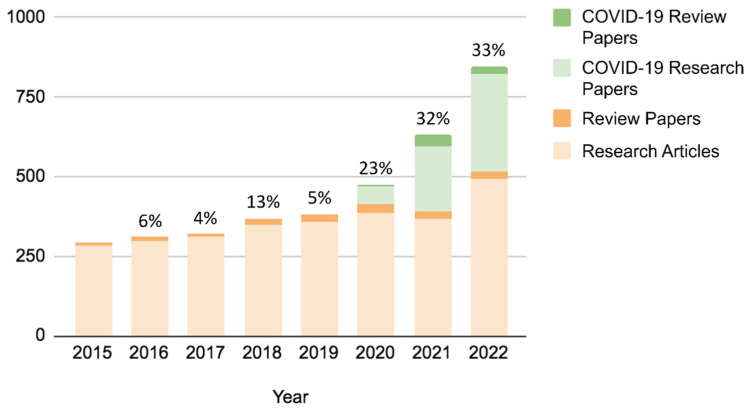
Trends in LAMP publications pre- and post-COVID-19 pandemic, analyzed through Scopus using keywords “LAMP” or “loop-mediated isothermal amplification.” A percentage increase from the immediate previous year is shown on top of each bar.

To understand how interest in LAMP grew as SARS-CoV-2 progressed, we used Scopus (Elsevier) to analyze the journal papers on LAMP pre- and post-pandemic. We first narrowed our search to journal publications between 2015 and 2019, searching keywords: “LAMP” or “loop-mediated isothermal amplification.” Results included the total number of research papers and review papers related to LAMP published in journals. Next, we conducted an identical search but switched our years of interest to 2020 through 2022. In the years preceding the pandemic, a steady growth (4–13%) in LAMP interest is seen (Figure 1). However, publications began to surge exponentially (23–33%) following the COVID-19 pandemic. In fact, in 2021 and 2022, over one-third of LAMP-centered publications were focused on SARS-CoV-2 testing (Figure 1).

As the need for extensive and rapid COVID-19 testing arose, researchers began investigating LAMP techniques. Efforts to optimize the accuracy and portability of LAMP techniques for SARS-CoV-2 point-of-care testing yielded improved specificity outcomes. In a systematic review and meta-analysis of COVID-19 LAMP platforms, multiple tests yielded comparable results to PCR, while maintaining lower costs and time to results [36].

Some of the frontrunners include: Lau et al., who used the Eiken Chemical Co. Loopamp RNA amplification kit paired with UV spectrophotometry [37]; Yan et al., who used the QIAamp Viral RNA Mini Kit to conduct RT-LAMP paired with real-time turbidity (visual) analysis, compared with the BGI PathoGenesis Pharmaceutical Technology commercial PCR kit [38]; Mohon et al., who used Warmstart^®^ Rtx Reverse Transcriptase and Bst 2.0 Warmstart^®^ DNA Polymerase paired with real-time fluorescence measurements [39]; and Chow et al., who developed a rapid colorimetric RT-LAMP assay, using reverse transcriptase, DNA polymerase, pH indicator, and six primers [40]. The results of these are shown below, in addition to the respective PCR results used in each study as a gold-standard comparison (Table 1):

As clearly shown, these research groups optimized LAMP assays to perform with a specificity and sensitivity similar to PCR, even at low viral loads. A common practice amongst these research groups was the use of highly specific primers to unique SARS-CoV-2 gene sequences (even compared to other SARS viruses), specifically regions on the S, E, orf3a, and orf1ab genes [37,38,39,40]. In addition, effective sample-collection methods helped maximize clinical sensitivity and specificity. In particular, nasopharyngeal swabs were found to be the preferable form of clinical testing [40].

**Figure 2:**
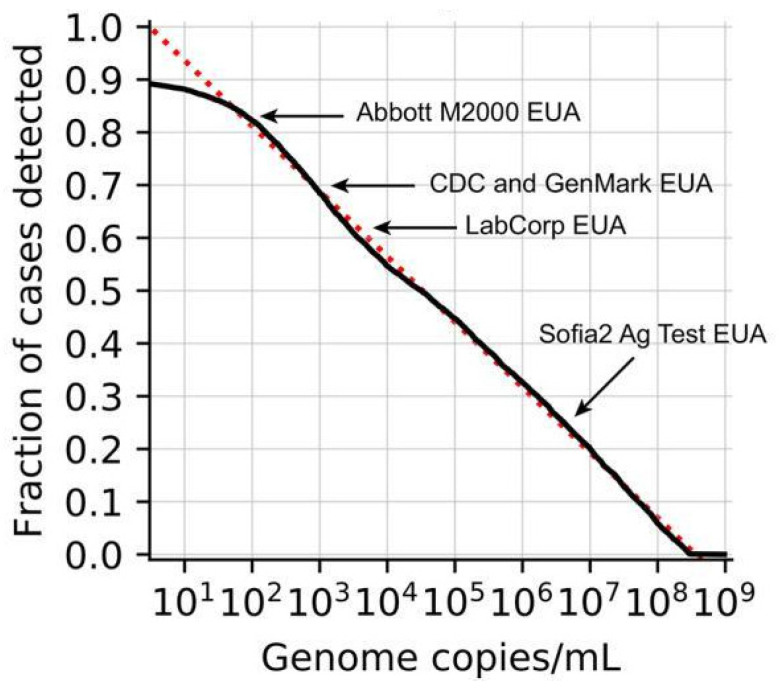
Fraction of SARS-CoV-2 detected are plotted against the LoD. Reprinted with permission from [41]. Copyright 2020, Arnaout et al., under Creative Commons Attribution 4.0 License.

Figure 2 shows the LoD, or analytical sensitivity, in relation to the true positive rate (=clinical sensitivity), for common commercial tests, where ~100 copies/mL is considered well-performing [41]. The LAMP results from the research groups above yielded LoD in the degree of 10 copies/mL, lower than the “optimal” LoD by 10-fold.

## 3. A Look into the Point-of-Care LAMP Market: Research and Commercial

In terms of point-of-care diagnostics, isothermal NAATs such as LAMP hold the most potential for portability, a crucial component of COVID-19 testing, and any diagnostic tests in more secluded areas. A significant obstacle to genetic identification at the point-of-care sites is initial DNA extraction, as the process is lengthy and requires skilled personnel to conduct lysis of the cell membrane, isolation and purification of the DNA, and various washing steps [42,43]. Some available techniques to speed up and simplify conventional RNA-purification and DNA-extraction methods include: microfluidic chips with cartridges dedicated to purification steps [44,45,46,47], bead- or membrane-based purification [48,49,50], and magnetic particles [51,52,53], which can bypass any centrifugation through components that directly bind to DNA/RNA for isolation [42]. These can speed up the process and lessen the amount of trained personnel needed. However, some LAMP techniques can entirely workaround “human-input” steps. Moehling et al. describe this as such: “target nucleic acids are made accessible by enzymatic (e.g., proteinase K), chemical (e.g., detergents), or physical (e.g., heat) lysis” [54]. In addition, SARS-CoV-2 research has yielded new success in direct LAMP testing (eliminating the need for DNA extraction or RNA purification) through optimization of primer design and reagent mixes [55,56]. With these methods, LAMP testing can be performed directly from collected samples (such as blood, saliva, etc.) regardless of complexity, with no preparatory steps needed.

Another obstacle in portable testing is the storage of reagents, many of which require freezing conditions. However, PCR or LAMP reagents can be lyophilized or air-dried, eliminating the need for freezing conditions to store reagents [57,58], which has already been employed by numerous research groups [59,60,61]. The actual amplification process of LAMP requires only a heat source, such as hot plates or even hand warmers. In addition, most detection techniques paired with LAMP are visual and can be paired with simple smartphone apps. Smartphone-based detection has been a common feature in point-of-care diagnostics, both before [62,63,64,65] and after [66,67,68] the pandemic. Considering the exceptional improvements of recent years, LAMP makes an excellent candidate for rapid point-of-care nucleic-acid testing: essential in both remote environments and situations that warrant mass testing [69,70,71,72,73,74,75].

Diving further into the current market paints a picture of the future of LAMP diagnostics. Presently, top players in commercial LAMP technology include: Thermo Fisher Scientific, Meridian Bioscience, Inc., HUMAN, New England Biolabs (NEB), and Eiken Chemical Co., Ltd., Tokyo, Japan [76]. Top products include kits and reagents (primers, extraction reagents, dyes, etc.) and detection technology (fluorescence and colorimetric color spectroscopy, and turbidity-measuring systems).

### 3.1. Leading LAMP Reagents

**Figure 3:**
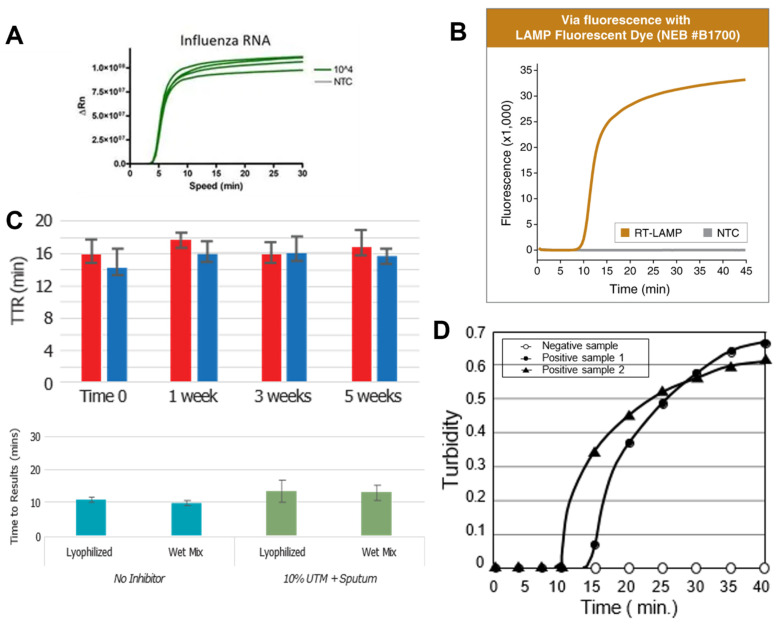
Leading LAMP reagents’ performance. (**A**) The reaction time of Thermo Fisher SuperScript™ IV RT-LAMP Master Mix for multiple pathogens. Reprinted with permission from [77]. Copyright 2022, Thermo Fisher. (**B**) Real-time NEB WarmStart^®^ LAMP kit (DNA & RNA) real-time and end-point detection and fluorescence & turbidity detection. Reprinted with permission from [78]. Copyright 2022, New England BioLabs. (**C**) The reaction times of Air-Dryable™ (top) and Meridian Lyo-Ready™ (bottom) kits are compared with conventional liquid reagents. Reprinted with permission from [79,80]. Copyright 2022, Meridian Bioscience^®^. (**D**) The reaction time of Eiken Chemical’s Loopamp kit. Reprinted with permission from [82]. Copyright 2022, Eiken Chemical Co., Ltd.

Regarding reagent kits, both Thermo Fisher Scientific and New England Biolabs (NEB) offer a LAMP master mix, which includes fluorescent dyes, but is compatible with any real-time or end-point detection (Figure 3A,B). Both offer rapid detection times (5–10 min in the case of Thermo Fisher and 10–15 min in the case of NEB) but require freezing conditions for storage [77,78]. NEB also provides colorimetric LAMP reagent kits, in which a change in pH resulting from the LAMP reaction turns the sample color from yellow to pink. This visual change does not require excitation by a fluorescent-light source, but the time to results is longer, from 15–40 min. Again, these reagents require freezing storage temperatures, limiting portability [79].

Meridian Biosciences Inc., on the other hand, offers two different reagent kits that can be stored at room temperature: Lyo-Ready™ LAMP/RT-LAMP Mixes and Air-Dryable™ LAMP/RT-LAMP Mixes (Figure 3C). As mentioned above, room-temperature reagent storage opens up far more opportunities in terms of portable and point-of-care LAMP testing. Meridian’s air-dried and freeze-dried reagents offer a similar detection time to the above kits [80,81].

Similarly, Eiken Chemical Co., Ltd. provides Loopamp detection kits: two of which (malaria- and tuberculosis-specific) have been approved for clinical use (Figure 3D). These kits can be paired with end-point fluorescence or real-time turbidity detection (due to both magnesium sulfate and calcein present in the mix), and the reagents can also be stored at room temperature. The time to results remains around 10–15 min [82]. Eiken Loopamp kits have been successfully used in lower-middle-income countries for remote point-of-care testing [83,84,85,86].

### 3.2. Leading LAMP-Detection Technology

The primary detection methods often paired with LAMP are (1) fluorescence changes, (2) colorimetric changes, and (3) turbidity changes. In fluorescence detection, considered the most sensitive of the methods, dyes are added to emit fluorescent signals upon amplification [87]. For example, calcein interacts with the magnesium byproducts from the LAMP reaction, emitting a strong, green fluorescence under visible light [88]. In addition, SYBR Green and Evagreen dyes are commonly used in this space, which can bind to the double-stranded DNA (LAMP products) and trigger a strong fluorescence emission [88]. However, these are nonspecific, and SYBR Green has been known to cause inhibitory issues when the concentration is too high. In colorimetric detection, added dyes change the solution color upon amplification, based on the pH changes and/or byproducts. For example, hydroxynaphthol blue (HNB) dye is commonly used, transforming from violet to blue due to magnesium byproducts [89]. Phenol red, found to be more sensitive than the former, changes from yellow to pink based on pH changes [89]. Colorimetric dyes offer simple detection with little chance of inhibition. However, they may be less specific than fluorescent dyes that can specifically bind to double-stranded DNA. Similarly, turbidity-based detection offers simplicity with no possibility of interference from dyes (since none are used). During LAMP reactions, magnesium-phosphate byproducts are produced, leading to turbid solutions, which can be used to monitor amplification [88]. Though the human eye can see changes in turbidity, these are far harder to differentiate; additional devices to quantify changes in turbidity are almost always used in these assays [89].

Though these methods produce visual changes and do not technically require equipment for detection, using devices to quantify these changes significantly improves accuracy and precision. In addition, devices can even offer real-time detection in the case of turbidity and fluorescence, considerably speeding up time-to-results. Numerous fluorescent-microplate readers and even real-time PCR machines originally designed for PCR can be applied to LAMP reactions. The same goes for real-time turbidity-monitoring systems. However, these tend to be expensive and defeat LAMP’s purpose of acting as a simpler and cheaper alternative to PCR. Some cheaper, handheld devices offer to quantify the end-point visuals of LAMP reactions.

In terms of price, colorimeters are the most affordable, followed by turbidimeters and, finally, fluorometers [90]. Looking at Hach, a well-known laboratory-equipment manufacturer, a field colorimeter (Hach DR900) costs USD 1491, a portable turbidimeter (Hach 2100Q) costs USD 1755, and a spectrofluorometer (Hach DR3900) costs USD 7779 [90,91]. Though, fluorometers alone can be slightly cheaper, as seen in the Qubit™ 4 Fluorometer by Thermo Fisher Scientific, which costs USD 3945 [92]. Regardless, the opposite trend is seen in terms of sensitivities: fluorometers are the most sensitive, followed by turbidimeters and colorimeters. In fact, fluorescence-based detection can be up to 1,000 times more sensitive than the other two absorption-based methods [87].

In order to reduce costs further, some research groups have worked to develop smartphone-based apps to quantify these parameters in NAAT detection. Color quantifying apps, such as “AssayColor”, are widely available and easily applied to colorimetric detection [93]. In addition, these can be applied to fluorescence detection, so long as an LED illuminates the samples. In 2014, a research group from the University of Sydney was one of the first to exhibit such a “makeshift” device [94]. The group used the Android application “Color Grab,” paired with a color filter over the smartphone’s own white light, in a 3D-printed device, to perform fluorescence quantification for the purpose of pH monitoring in water samples [94]. The device produced comparable results to a spectrophotometer, while maintaining only a fraction of the costs [94]. Similarly, in 2019, a research group at the University of California, Los Angeles, developed a smartphone-based turbidity analyzer [95]. This device costs only USD 45 to produce and offers similar accuracy to benchtop turbidimeters. The most promising detection devices in terms of portability, affordability, and rapidity are seen in smartphone applications in emerging research. Since smartphone access has become widespread, these platforms offer more reliable detection from visual changes in LAMP reactions, all for generally under USD 100.

**Figure 4:**
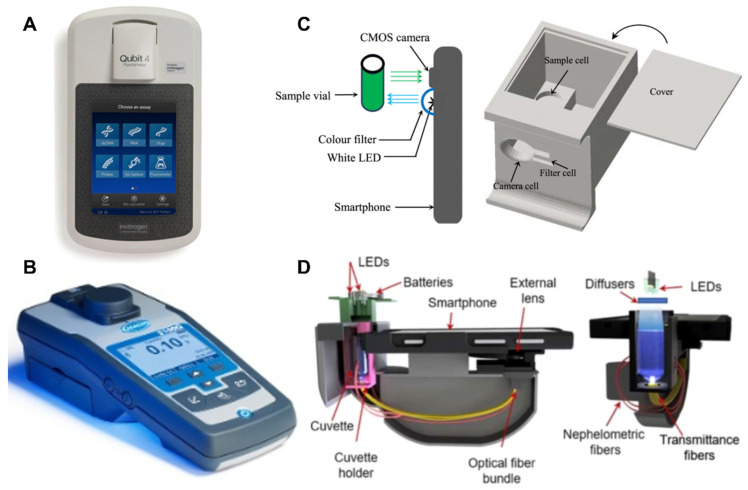
Leading detection devices. (**A**) Thermo Fisher Scientific Qubit™ 4 Fluorometer. Reprinted with permission from [92]. Copyright 2022, Thermo Fisher. (**B**) Hach 2100Q Portable Turbidimeter. Reprinted with permission from [91]. Copyright 2022, Hach. (**C**) Fluorescence-measurement device: setup and performance. Reprinted with permission from [94]. Copyright 2022, IEEE. (**D**) Smartphone-based turbidimeter platform: configuration and performance. Reprinted with permission from [95]. Copyright 2019, Koydemir et al., under Creative Commons Attribution 4.0 License.

Figure 4 summarizes the leading LAMP-detection devices and their performances.

### 3.3. Leading “All-In-One” LAMP Systems

**Figure 5:**
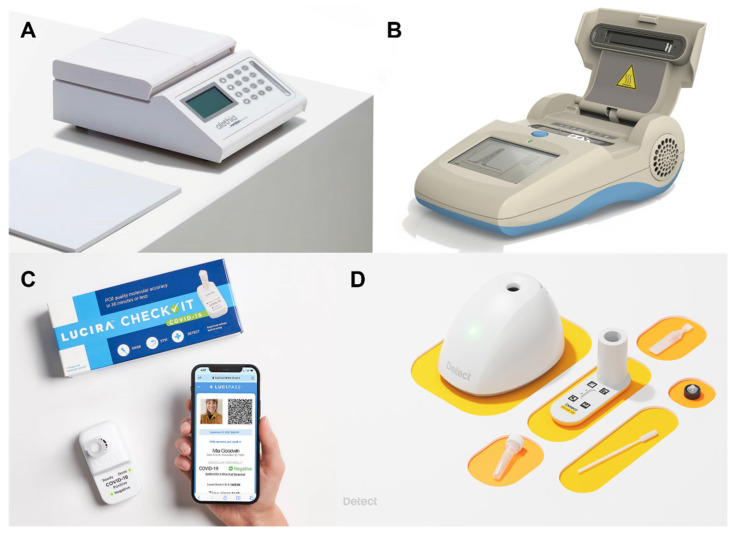
Commercial LAMP devices. (**A**) Alethia portable LAMP platform (top) and DNA extraction prep (bottom). Reprinted with permission from [96]. Copyright 2022, Meridian Bioscience^®^. (**B**) Genie III portable LAMP platform. Reprinted with permission from [102]. Copyright 2022, OptiGene. (**C**) Lucira™ Check It COVID-19 Test Kit. Reprinted with permission from [103]. Copyright 2022, Lucira. (**D**) Detect COVID-19 Starter Kit™. Reprinted with permission from [104]. Copyright 2022, Detect.

Several LAMP devices have been approved for clinical use. We will discuss the targets, detection methods, and performance metrics (specificity and sensitivity) of these devices in this section. Meridian Bioscience advertises its Alethia line (previously known as Illumigene), which detects a variety of pathogen targets, from human papillomavirus (HPV) to *Streptococcus* spp., through real-time turbidity measurements [96,97]. DNA extraction is conducted through a simple centrifuge-free filtration system [98,99]. The *Mycoplasma pneumoniae* Alethia platform provides the results within an hour, with 97.7% clinical specificity and 96% clinical sensitivity, for USD 5170 [99]. The product is portable, while offering similar accuracy to that of PCR (Figure 5A).

Another LAMP device commercially available is OptiGene’s Genie III, which conducts real-time fluorescence monitoring (Figure 5B). A research group in Guinea used the Genie III for an Ebola LAMP assay, which yielded 100% (95% CI: 93.3–100) clinical specificity and 97.9% (95% CI: 88.7–100) clinical sensitivity [100]. Unfortunately, despite decent accuracy and portability, the Genie III still requires DNA extraction steps and is incredibly costly at USD 19,090.90 [101,102].

Other “all-in-one” LAMP devices created during the COVID-19 pandemic include Lucira™ [103] and Detect™ [104] (Figure 5C,D). Both are designed for at-home testing by the patient. The kits are designed for self-administered nasal swabs, and the results are communicated through a smartphone app. Both devices are small, about the size of a cup. Lucira costs USD 75 for a single-use kit, offering clinical sensitivity of 92% and clinical specificity of 97%. Detect™, on the other hand, costs USD 85 for a single test plus a reusable “hub.” Clinical sensitivity is slightly lower, at 90.9%, but clinical specificity is higher, at 100%. Specifications of these commercial LAMP devices are compared below (Table 2).

### 3.4. Recent Research on All-In-One LAMP Systems for SARS-CoV-2

**Figure 6:**
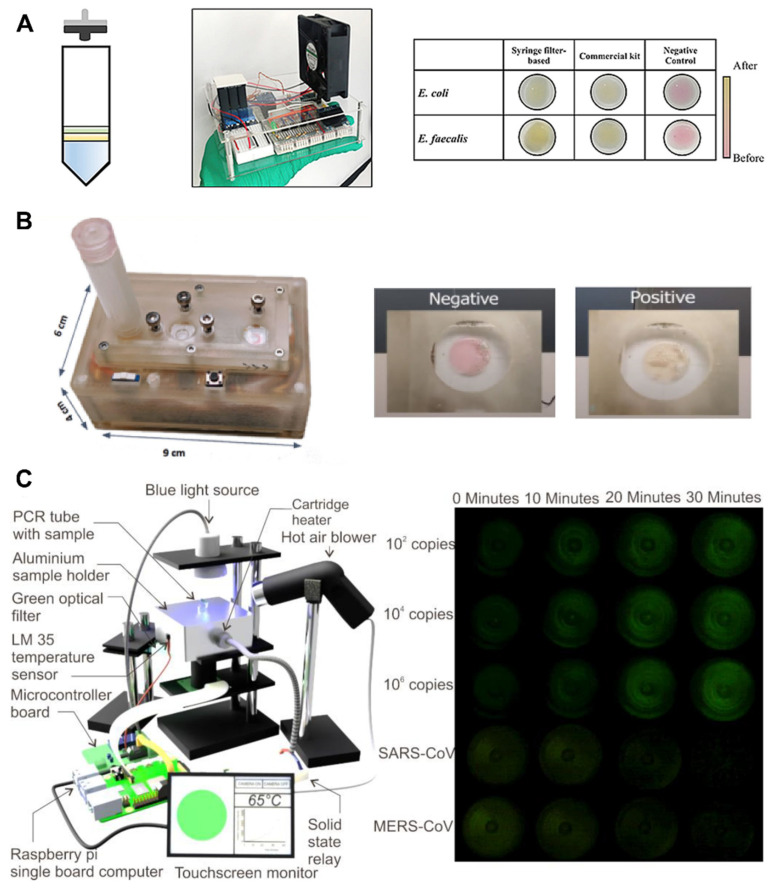
LAMP devices in recent research. (**A**) Lee et al.’s portable LAMP device. Reprinted with permission from [105]. Copyright 2022, Elsevier. (**B**) Deng et al.’s portable LAMP device. Reprinted with permission from [106]. Copyright 2021, Deng et al., under Creative Commons 4.0 Attribution License. (**C**) Sreejith et al.’s portable COVID-19 LAMP device. Reprinted with permission from [107]. Copyright 2021, Sreejith et al., under Creative Commons 4.0 Attribution License.

Promising LAMP platforms can also be seen in emerging research, especially, in recent years, in response to the COVID-19 pandemic. In 2019, a research group from the National University of Singapore developed a smartphone-based colorimetric LAMP device that yields results within an hour. The system utilizes a syringe filter with magnetic beads for rapid DNA extraction and is 10 times more specific than PCR methods (Figure 6A) [105].

More recently in 2021, a research group from Monash University developed a small, portable SARS-CoV-2 LAMP device based on colorimetric detection (Figure 6B) [106]. Nasal samples are loaded into a disposable cartridge, and the time to results is merely 35 min. In addition, the platform eliminates lengthy and complex DNA-extraction methods by simply boiling samples upon insertion into the “lysing chamber” of the cartridge. The analytical sensitivity of the assay is average, with an LoD of 300 copies/mL.

Additionally, in 2021, a research group from Griffith University fabricated a portable LAMP device targeting SARS-CoV-2 based on fluorescence emission (Figure 6C) [107]. According to Sreejith et al., the system (costing USD 100) consists of an “aluminum block embedded with a cartridge heater,” a “single-board computer and camera,” and an LED. Time to results is as low as 10–20 min, and the LoD is 100 copies/mL.

**Figure 7:**
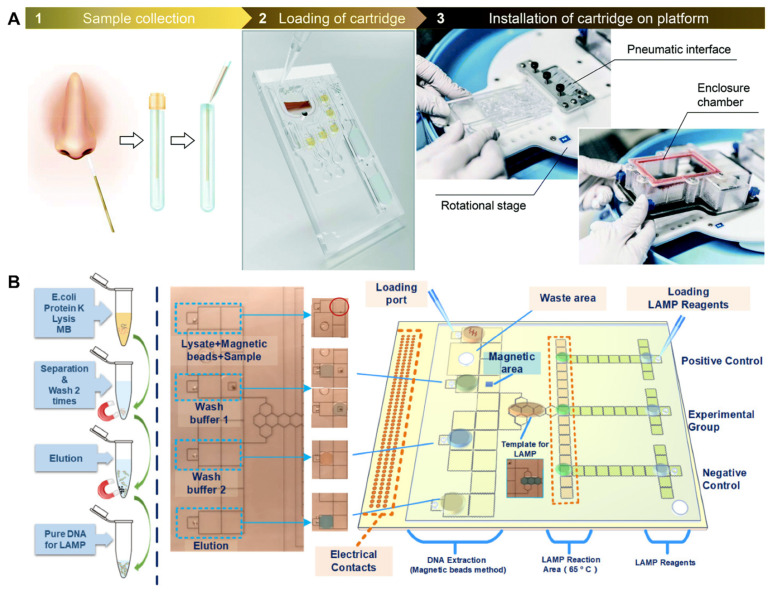
All-in-one microfluidic LAMP systems for COVID-19 detection. (**A**) Malic et al.’s centrifugal microfluidic LAMP system for COVID-19 detection. Reprinted with permission from [108]. Copyright 2022, Royal Society of Chemistry. (**B**) Hu et al.’s digital microfluidic LAMP system for COVID-19 detection. Reprinted with permission from [109]. Copyright 2022, Hu et al., under Creative Commons 4.0 Attribution License.

A more sophisticated all-in-one LAMP can also be conducted on microfluidic platforms. For example, Malic et al. [108] designed a centrifugal microfluidic system for detecting COVID-19 with RT-LAMP, as shown in Figure 7A. Hu et al. [109] developed a digital microfluidic system for detecting COVID-19 with RT-LAMP, as shown in Figure 7B. Droplets were manipulated over an array of electrodes in a programmable manner, hence, “digital” microfluidics, to conduct several different reactions necessary for RT-LAMP. Such microfluidic platforms have been demonstrated for detecting numerous other bacterial and viral pathogens, using both LAMP and PCR. Hence, it is a matter of time to demonstrate the same for COVID-19. Such microfluidic systems provide true all-in-one and programmable features at the cost of device complexity (compared to the current commercial systems).

## 4. LAMP Potentials Disregarded in COVID-19 Testing

Interest and improvements in the world of LAMP have been clearly impacted by the COVID-19 pandemic and the necessity for rapid, portable, and cheap diagnostic testing. In 2020, at the start of the pandemic, multiple research groups studied LAMP SARS-CoV-2 testing and reported lower accuracy than in the ‘gold-standard’ PCR. Likely, this played a role in PCR and rapid antigen tests dominating the public market. Kortela et al. reported a decent overall clinical sensitivity of 89.9% with RT-PCR for clinical SARS-CoV-2 samples [110]. In October of 2020, Österdahl et al.’s investigation of RT-LAMP compared to RT-PCR resulted in a PPV (positive predictive value) of 73% and NPV (negative predictive value) of 80%, meaning both clinical sensitivity and specificity were lower than that of PCR, though the test is especially prone to false positives [111]. Artik et al. also reported high false positives in their LAMP reactions (with a 63% positivity) [112]. Two groups, Usherwood et al. and Pu et al., reported LAMP tests that yielded lower clinical sensitivities, resulting in higher false-negative rates [113,114]. However, Usherwood et al. explain that false positives may have been investigated less regarding COVID-19, since these are of less concern than false negatives [113].

However, LAMP still outperformed (as shown in Table 1 and Table 2) the rapid antigen tests in accuracy, as the latter yielded a clinical sensitivity of 65% (58% in some cases) [115,116,117,118]. In addition, breakthrough LAMP assays arose throughout the pandemic. As previously mentioned, a few research groups even produced results comparable to PCR in their SARS-CoV-2 LAMP assays [36,37,38,39,40]. Experts have been working towards eliminating non-specific amplification and increasing overall accuracy in LAMP diagnostics for years. These efforts were only magnified through COVID-19 research [119,120,121].

**Figure 8:**
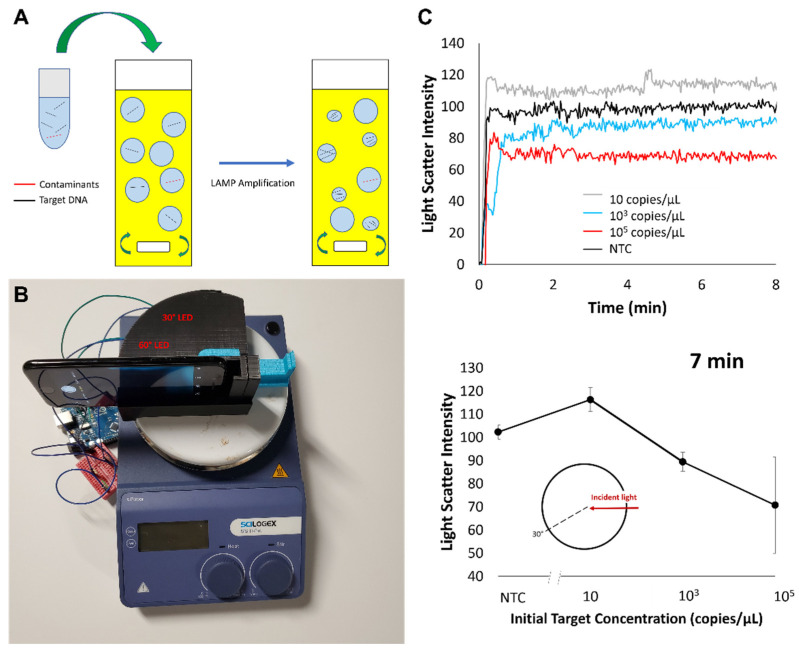
Day et al.’s emulsion LAMP. (**A**) Compartmentalization of emulsion LAMP reactions. Reprinted with permission from [32]. Copyright 2021, Day et al., under Creative Commons 4.0 Attribution License. (**B**) Smartphone detection setup. Reprinted with permission from [123]. Copyright 2022, Elsevier. (**C**) Smartphone detection results. Reprinted with permission from [123]. Copyright 2022, Elsevier.

Various methods, such as the addition of DMSO (dimethyl sulfoxide) [33,34,35] to the reaction mix and the use of OSD (oligonucleotide strand displacement) probes [24,25,26,27,28], have been proven to improve LAMP accuracy. In particular, compartmentalization of the reaction through microfluidics [29,30,31] and emulsion [32,122,123] techniques have shown substantial success in improving accuracy by limiting the effects of contaminants. This has been applied to PCR reactions as well, most popularly through digital-droplet PCR (ddPCR), in which reactions are partitioned (using oil) into thousands of “droplets,” eliminating interference from reaction-inhibiting particles [124]. Droplets can be formed within the microfluidic channels in small, aqueous plugs, each separated by the oil phase, i.e., microfluidic-based compartmentalization. Alternatively, they can be created via water-in-oil emulsion using surfactants, i.e., emulsion-based compartmentalization. Compartmentalization can also be achieved by partitioning the liquid into a micro- or nanowell or an array of miniaturized chambers. Software embedded into thermocyclers can calculate the ratio of positive droplets (with fluorescent emission) and negative droplets to quantify the concentration of target nucleic acids in the sample [124]. However, this demands more sophisticated and expensive equipment than that of emulsion LAMP. Day et al. designed an emulsion-LAMP setup for SARS-CoV-2 detection, using a simple Arduino circuit, two LEDs, and a smartphone that reported 100% specificity with an LoD of 10 copies/mL (analytical senstivity) (Figure 8) [98]. In this simple-yet-effective setup, the Arduino rapidly switches each LED on and off, while the phone captures change in the light scatter of the reaction through a timelapse that is later analyzed in Python code [123]. Day et al.’s smartphone-emulsion-LAMP setup and performance results are shown below. Again, the potential for portable, rapid, and highly accurate point-of-care LAMP diagnostics has clearly emerged from COVID-19 research.

## 5. Conclusions

In a sense, the coming together of scientists across the globe for a common cause could be considered one positive that arose from the COVID-19 pandemic. With a vast number of great minds at work, the field of both diagnostics and vaccines saw rapid improvements and developments. LAMP is no exception. As efforts rose to control the virus through frequent testing and tracking, cheap, fast, and effective tests became necessary. Rapid antigen tests provided a quick time to results, with wait times around 15 min, while suffered from unreliable results as described in Section 4. The spotlight for gold-standard accuracy in SARS-CoV-2 tests quickly shone on PCR. However, especially with rising demand, the limited laboratory equipment needed for PCR testing created severe delays. At this point, results would be essentially useless since a positive status can change so quickly. The need for rapid and accurate yet cost-effective SARS-CoV-2 tests promptly arose with interest in LAMP.

LAMP offers the accuracy of a nucleic-acid test with the simplicity and cost of an isothermal test. Research groups have developed rapid LAMP systems for point-of-care use with increasing efforts toward portability and creating remote-friendly tests. As a result, conventional, lengthy DNA-extraction methods have been avoided; reagents have been lyophilized, eliminating freezing storage temperatures; and even small-sized detection devices have surfaced.

Though LAMP is known for its shortcomings in sensitivity and specificity, the accuracy is still far superior to that of the rapid antigen test. At the very least, the method offers a middle ground to the unreliable results of rapid antigen tests versus the prolonged time to results of PCR tests. Not to mention, progress in LAMP research due to the COVID-19 pandemic yielded some tests with comparable sensitivity and specificity results to that of PCR. A comparison of existing, laboratory-based LAMP and PCR methods for COVID-19 detection has recently been summarized by Pu et al. [117]. They mentioned that the false rates are substantial, 6–12%, for both LAMP and PCR. Yet, still, LAMP was rarely embraced or offered in testing locations at the earlier phases of the COVID-19 pandemic.

Possibly, these advancements came to light too far into the pandemic. As testing systems were already well-established, any disruptions may have seemed more of a hassle than a benefit. However, the old stigma around LAMP diagnostics may also still reign. Perhaps it is time, as a scientific and medical community, to re-evaluate the perceptions of loop-mediated isothermal amplification as a whole and consider the progress and advantages of this method.

## Figures and Tables

**Table 1:** Top-performing LAMP diagnostic tests for SARS-CoV-2.

Research Group	Clinical Sensitivity (%)	Clinical Specificity (%)	Total Samples Tested	LoD (Copies/mL)
Lau et al. [37]	100	100	89	1
Yan et al. [38]	100	100	130	20
Mohon et al. [39]	98.5	100	124	25
Chow et al. [40]	98.2	100	366	42

**Table 2:** Specifications of commercial LAMP devices. Data retrieved from [97,98,99,100,101,102,103,104].

	Alethia	Genie III	Lucira	Detect
Dimension	A4 paper	250 × 165 × 85 mm	A cup	A cup
Sample type	Extracted DNA	Extracted DNA	Nasal swab	Nasal swab
Assay time	1 h	15 min	30 min	1 h
Clinical sensitivity	96.0%	97.9%	92%	90.9%
Clinical specificity	-	100%	97%	100%

## Data Availability

Not applicable.
